# Microfluidic-assisted Formation of Highly Monodisperse and Mesoporous Silica Soft Microcapsules

**DOI:** 10.1038/s41598-017-16554-4

**Published:** 2017-11-27

**Authors:** Nizar Bchellaoui, Zain Hayat, Mohamed Mami, Rachida Dorbez-Sridi, Abdel Illah El Abed

**Affiliations:** 1 Laboratoire de Photonique Quantique et Moléculaire, CNRS-UMR 8537, ENS-Paris Saclay, CentraleSupélec, 61 avenue du Président Wilson, 94235 Cachan, Cedex France; 2 Laboratoire de Physico-chimie des Matériaux, University of Monastir, Monastir, Tunisia

## Abstract

The fabrication of mesoporous silica microcapsules with a highly controlled particle size ranging in the micrometer size presents a major challenge in many academic and industrial research areas, such as for the developement of smart drug delivery systems with a well controlled loading and release of (bio)active molecules. Many studies based on the solvent evaporation or solvent diffusion methods have been developed during the last two decades in order to control the particle size, which is often found to range at a sub-micrometer scale. Droplet-based microfluidics proved during the last decade a powerful tool to produce highly monodisperse and mesoporous silica solid microspheres with a controllable size in the micrometer range. We show in the present study, in contrast with previous microfluidic-assisted approaches, that a better control of the diffusion of the silica precursor sol in a surrounding perfluorinated oil phase during the silica formation process allows for the formation of highly monodisperse mesoporous silica microcapsules with a diameter ranging in the 10 micrometer range. We show also, using optical, scanning and transmission electron microscopies, small angle x-ray diffraction and BET measurements, that the synthesized mesoporous silica microcapsules exhibit a soft-like thin shell with a thickness of about 1 *μm*, across which 5.9 nm sized mesopores form a well-ordered hexagonal 2D network. We suggest and validate experimentally a model where the formation of such microcapsules is controlled by the solvent evaporation process at the droplet-air interface.

## Introduction

Well-ordered mesoporous silica materials with a controllable nanopore size and structure are of a significant importance in many academic and industrial research areas. More particularly, mesoporous silica microspheres and microcapsules have potential applications in drug-delivery^1–3^, catalysis^4^, biosensing^5^ and/or tissue bio-engineering^6^, see for a detailed review refs^7,8^. The fabrication of such microparticles relies mostly on methods derived from the so-called ESE (Emulsion Solvent Evaporation) approach, developed a decade ago by Andersson *et al*.^9^, which is based on the combination of the well-known Stöber sol-gel technique^10^ and the dispersing in a continuous oil phase of a silica precursor solution (sol) droplets. The latter consists generally on a solution of tetraethyl orthosilicate (TEOS) or tetra methoxy orthosilicate (TMOS), a mixture of water/alcohol solvents and meso-structuring surfactants (generally a PEG derivative or CTAB). Their formation is enabled by the progressive diffusion of the alcohol solvent component from sol droplets towards the continuous oil phase. This leads in turn to a self-assembling of the meso-structuring surfactant molecules inside droplets and the formation of spherical or cylindrical micellar structures around which silica solidifies^11^. Also, since water does not readily diffuse into the oil phase, the ESE method requires usually further evaporation of water at elevated temperature and/or reduced pressure.

The synthesis of hollow mesoporous silica microspheres (HMSs) can be achieved following the ESE method and the addition of either soft or hard sacrificial templates on which silica solidifies and forms the mesoporous shell. Ultimately, such templates should be removed by thermal or chemical treatments enabling thus the formation of a hollow interior inside the microparticles, see for a detailed review ref.^8^. One should note however that compared to soft templates, hard templates were shown to be more effective for synthesizing HMSs with defined particle size and morphology as demonstrated for instance by Qi *et al*.^12^. These authors used latex nanoparticles to achieve highly monodisperse sub-micrometer sized HMSs where the particle size and the mesoporous shell thickness could be fine-tuned by adjusting the amount of latex templates and silica source. Nevertheless, the general preparation and removal procedures of the sacrificial templates are often complicated, uneconomic, and time consuming. Another major limitation of the standard soft template method is the rather broad size distribution of the as-synthesized microspheres, owing to the poor control of the distribution size of the templating droplets, see for a review ref.^7^. Therefore, it is highly valuable to develop a soft template method which allows for the synthesis of highly monodisperse mesoporous hollow silica microcapsules with a size ranging in the micrometer range and allowing for a large volume of the inner chamber.

Actually, Soon after the pioneering work of Andersson *et al*.^9^, droplet-based microfluidics^13,14^ has proven to be a straightforward and robust approach to address at the microscale the size distribution of the ESE as-synthesized silica microspheres, as shown first by Caroll *et al*.^15^. These authors achieved the fabrication of highly monodisperse silica microspheres from monodisperse sol droplets generated in a microfluidic device and condensed quickly in a flask outside the microfluidic device. Later, Lee *et al*.^16^ developed a one-step microfluidic approach, which enables for a rapid *in-situ* diffusion of the sol solvents in the carrier oil (hexadecane) and a rapid condensation of silica microspheres in the microfluidic device. Thus, droplet-based microfluidics proved a powerful tool to address not only the size distribution of the ESE as-synthesized MSMs but also their shape and their surface morphology, since such features are controlled by two dynamic processes: (i) the diffusion of the precursor solution from the droplets to the carrier oil phase and the formation of an interfacial subphase, and (ii) a fast diffusion of alcohol from the interfacial subphase into the continuous oil phase. Hence, in order to reduce the rather long time required for silica condensation, Chokkalingam *et al*.^17^ developed another one-step approach, which enabled to achieve highly mesoporous silica microspheres with a large specific surface area, ca. 820 *m* ^2^/*g*, by triggering a quick condensation of sol droplets inside the microfluidic channels by means of electrodes embedded inside the microfluidic device. Noteworthy, Chokkalingam *et al*. obtained relatively small silica microspheres with a mean size of about 3.5 *μm* and a mass density of about 0.32  *g*/*cm* ^3^, by regards to the used silica precursor concentration (1.5 M) and the initial size of droplets (115 *μm*). Also, one should mention that, in contrast with Caroll’s^15^ and Lee’s^16^ works, Chokkalingam *et al*. used a perfluorinated oil (namely, perfluodecalin), but with a high concentration of stabilizing droplets surfactants, i. e., 20% (w/w), in order to stabilize droplets against merging. This may explain why, though perfluorinated oils are known to solubilize none of the sol components, one could observe a large difference between the amount of silica brought initially in microdroplets (in the form of TMOS) and the final amount of silica remaining in the silica microspheres, ~10^−7^ g and ~10^−12^ g, respectively. In such conditions, indeed, the carrier oil contains such a large number of surfactant molecules and micelles that a large proportion of the droplets content could become soluble in the oil phase.

In contrast with previous microfluidic approaches, we used a reduced silica precursor concentration (0.34 M) and a perfluorinated oil, as a surrounding organic phase where none of the sol droplets contents is soluble. This approach allows for a better control of the mass transport process from droplets towards the surrounding media. This assumption is particularly valid in our study as we used an oil-soluble droplet-stabilizing surfactant (TB-Krytox) with a concentration as low as 1% (w/w). This concentration is for instance 20 times smaller than the one used by Chokkalingam *et al*.^17^ and it allowed for the reducing of the diffusion and the dispersion of both solvent and sol while droplets flow along the microfluidic channel and tubing.

Highly monodisperse microdroplets, which were generated in a microfluidic device, were used as soft templates to synthesize highly monodisperse silica microcapsules with a diameter of 10 *μm* and a mesoporous shell thickness of about 1 *μm*. The synthesized microcapsules exhibit a well-ordered nanopores structure and a soft mechanical feature. Owing to the flexibility offered by the microfluidics approach, the size and the narrow size distribution of the synthesized microcapsules may be easily optimized. We anticipate that such microcapsules may have very promising applications in quantitative mass transport and bio-encapsulation studies. Our approach allows also for a quantitative analysis of the dynamics of the formation of mesoporous silica microcapsules and for the characterization of their structural features such as the mass density and the thickness of the silica shell. We suggest a model where their formation is controlled by the solvent evaporation process at the droplet-air interface.

## Results and Discussion

### Collection and condensation of sol droplets

**Figure 1:**
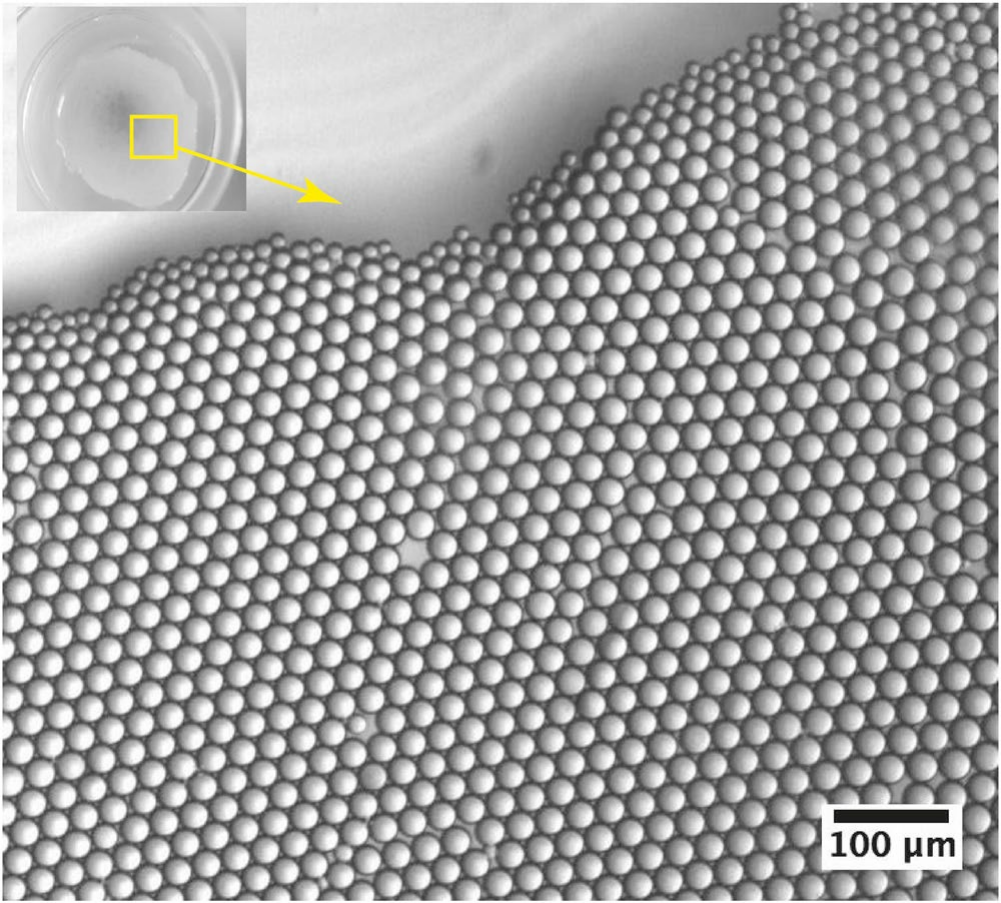
Optical micrograph of a floating monolayer of silica precursor microdroplets at the oil-air interface, in a Petri dish, which reach a size of 26.5 *μm* after 2 hours from the beginning of the collection and the drying processes. The initial size of microdroplets was 30 *μm*.

**Figure 2:**
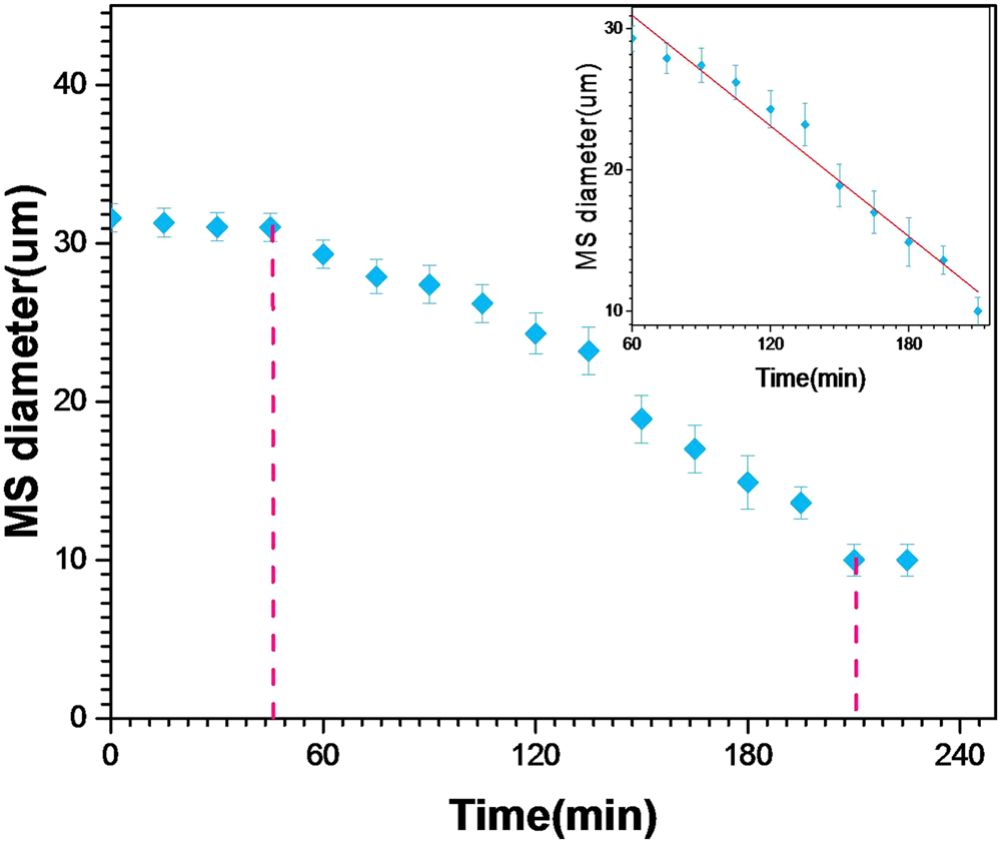
Change of sol microdroplets diameter versus time, as determined from image analysis of condensing microdroplets at the oil-air interface.

Microdroplets were collected in a Petri dish where they form a monolayer at the oil-air interface, as perfluorinated oil possesses a greater density (*d*_*HFE*−7500_ = 1.62) than the sol phase, as illustrated in Fig. 1. The condensation of microdroplets starts significantly and practically one hour after the droplets have reached the oil-air interface at the Petri dish collector, as shown on Fig. 2. Noteworthy, the condensation of silica droplets in our study is mainly controlled by the slow evaporation process of the solvent at the oil-air interface. This is particularly visible from Fig. 1, where droplets located at the edge of the monolayer appear smaller than those located in its inner part, where the solvent evaporation takes place only through the droplets upper side (in contact with air). Whereas for droplets located at the external part of the droplets monolayer, evaporation of the solvent takes place through both the upper side and the lateral side of the droplets. It is also interesting to note that once the droplets start to condense, their size decreases linearly versus time, as shown by the linear fit in the inset of Fig. 2, before they reach a final size of about 10 *μm* after a period of about 2.5 hours.

The linear decrease of the droplets size may be explained easily according to the following model. Let’s consider a droplet with a radius *R* at a given time *t* and let us assume that the droplet volume rate, $\frac{dV}{dt}$, due to the solvent evaporation, is proportional to the surface of the droplet in contact with air, that is approximately 2*πR* ^2^:

1 $$\frac{dV}{dt}=4\pi {R}^{2}\frac{dR}{dt}\propto -{R}^{2}$$

Integration of equation eq. 1 leads to:

2 $$R(t)=-{K}_{1}t+{K}_{2}$$

where *K*_1_ and *K*_2_ are arbitrary constants. The observed experimental *K*_1_ = −1.1 (*μ*m/min) value should be related to the surface density of pores through which the solvent evaporates.

### Mesoporous silica microcapsules characterization

**Figure 3:**
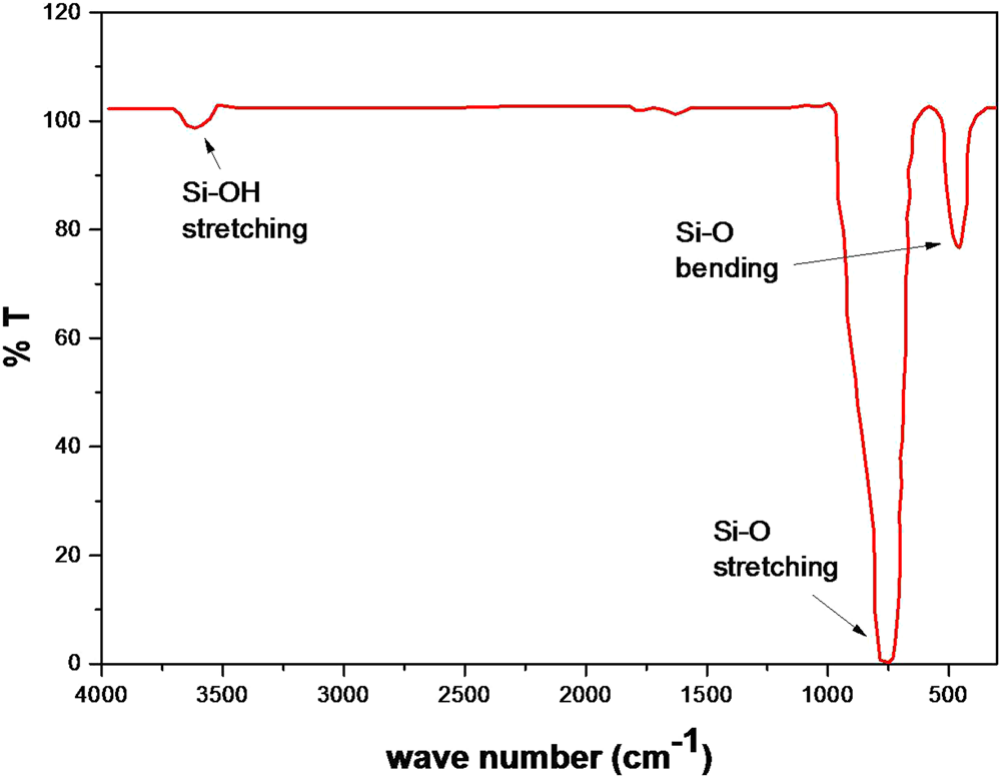
FTIR spectra of synthesized silica microspheres which exhibit absorption bands of silicates only.

After they have reached their final size (≃10 *μm*), silica microparticles were left at room temperature under normal pressure during 24 hours and were desiccated at a temperature equal to 150 °C during another 24 hours. Samples were then characterized using FTIR, scanning and transmission electron microscopies (SEM and TEM), fluorescence microscopy, BET (Brunauer, Emmett, and Teller) and Small Angle X-ray diffraction (SAX) techniques. Figure 3 reports FTIR spectrum of our samples, performed in the 400–2000 cm^−1^ spectral range, after the drying process at 150 °C. It shows only typical absorption bands of the silicate vibrations at 467, 798, 3600 cm^−1^, which are usually assigned to Si-O bending mode, Si-O symmetric stretching mode and Si-OH symmetric stretching mode, respectively. No absorption bands of the surfactant molecules could be observed, which indicates that the surfactant molecules have been eliminated during the dessication process.

**Figure 4:**
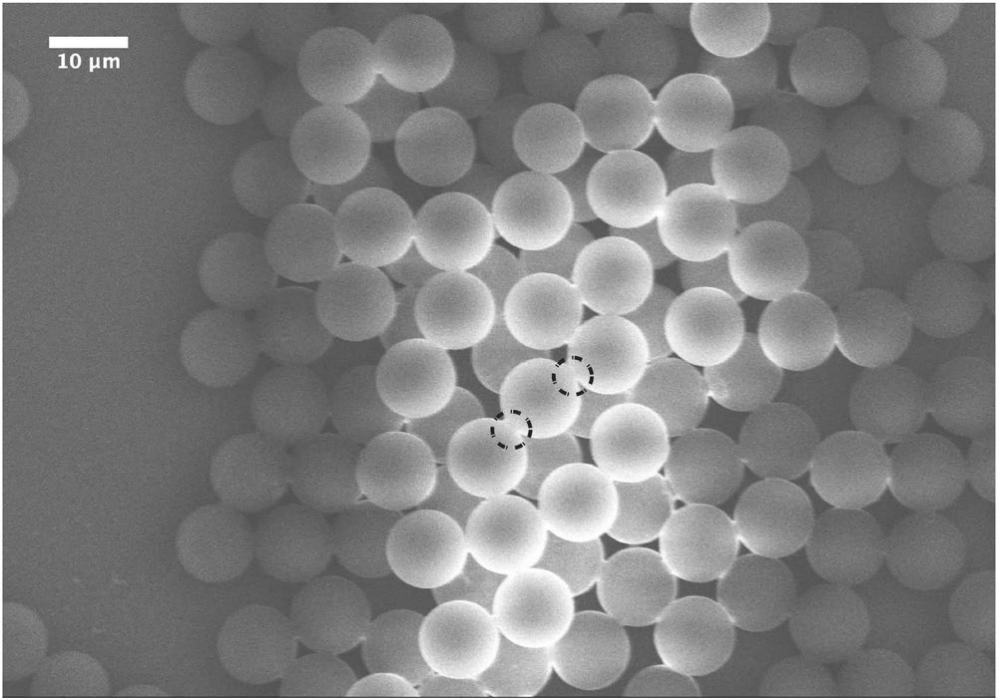
SEM micrograph of highly monodisperse silica microspheres obtained from 30 *μm* sized silica precursor microdroplets. Silica microspheres appear to interact strongly with their neighbors to from overlapping regions at their contacts, emphasized by the black dashed circles.

**Figure 5:**
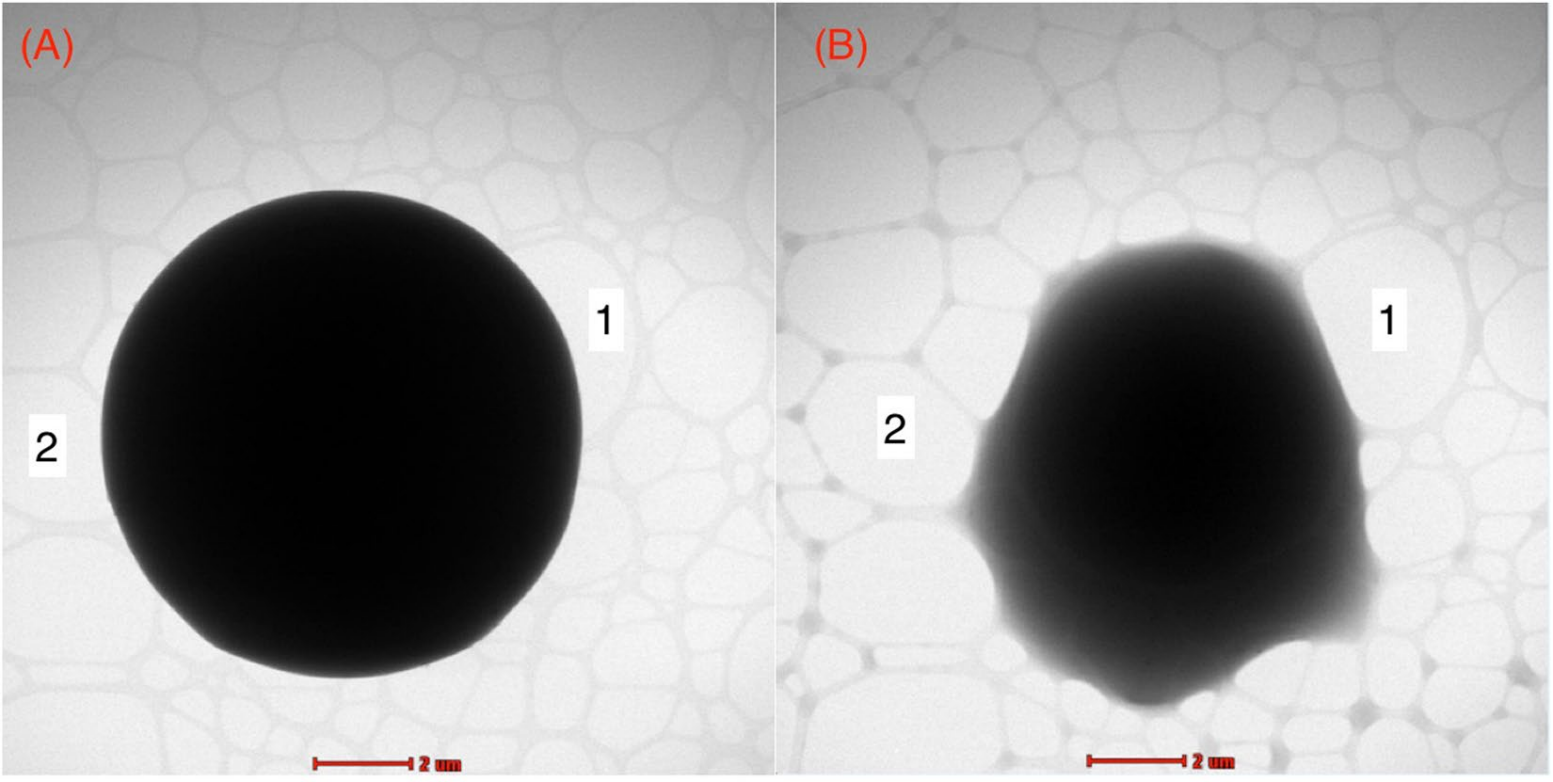
TEM micrograph of 10 *μm* silica microcapsules which shows the soft and hollow feature of the synthesized microspheres. Silica microparticles were deposited on a thin carbon membrane of the TEM grid. Numbers (1 and 2) were added as milestones to help following the displacement of the microcapsule on the two micrographs during the collapse process under the effect of the incident electron beam and few seconds of irradiation at 200 kV.

**Figure 6:**
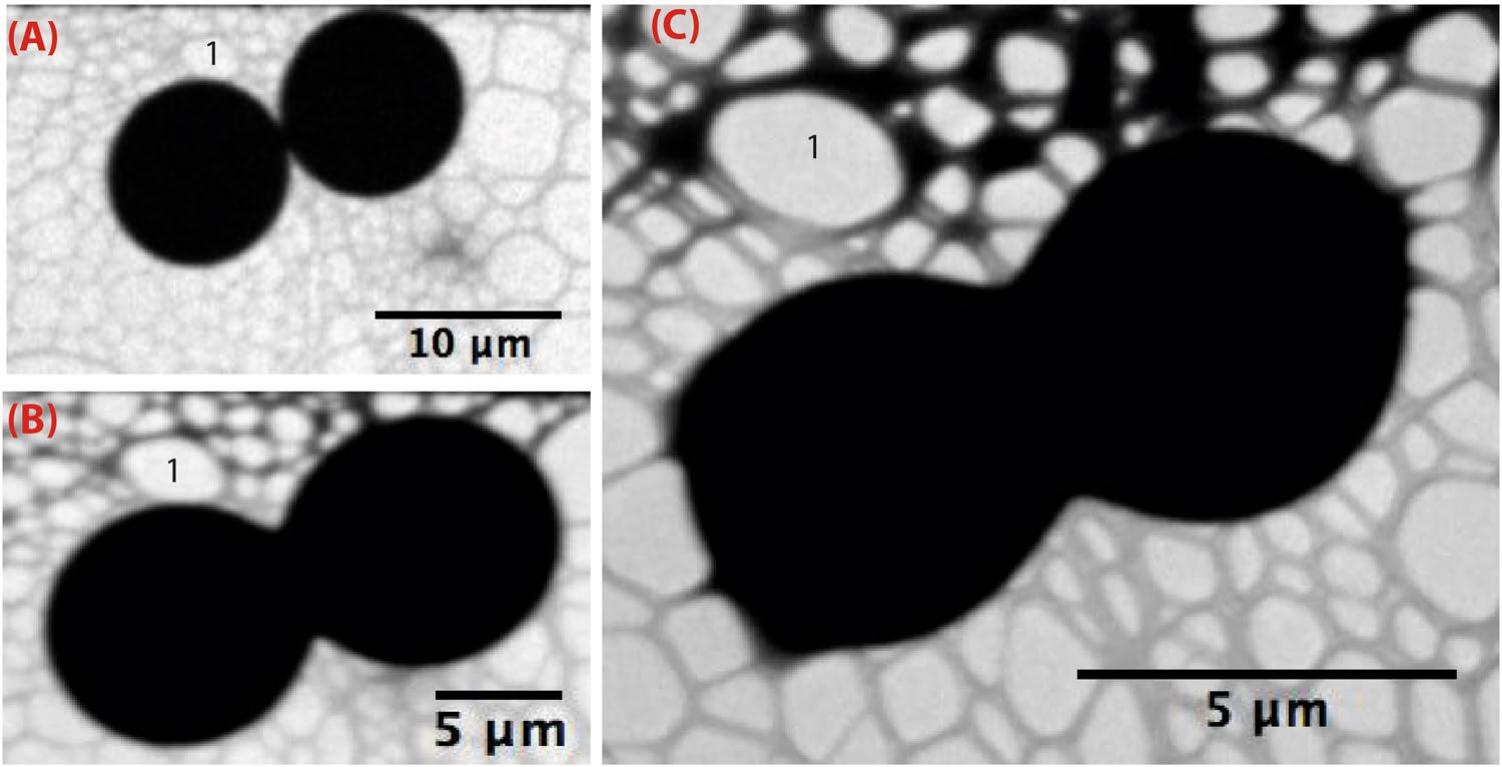
TEM micrographs showing the merging of neighboring silica microcapsules under the effect of the incident electron beam.

Figure 4 shows a typical SEM image of silica microspheres obtained from silica precursor droplets with a mean diameter *D*_*drop*_ = 30 *μm* (±1 *μm*). As one may remark, highly monodisperse silica microspheres with a typical size *D*_*p*_ = 10 *μm* (±0.2 *μm*) are obtained. Moreover, considering the initial sol concentration (0.34 M) and the final size of the silica microspheres, one may deduce easily a mass density of about *ρ*_*p*_ = 0.55 g/cm^3^ for these microspheres. The later value could be accepted by regards to the standard value of the mass density of non mesoporous silica, i. e., 2.2 g/cm^3^. Figure 4 shows also that silica microspheres interact strongly and form overlapping regions at their contacts, as emphasized by the black dashed circles in Fig. 4. This feature seems to indicate that microspheres have a soft-like structure, as confirmed by TEM images shown in figures Figs 5 and 6.

Because of their large size, by regards to the thickness of samples which may analyzed with TEM, i.e., few hundreds nanometers thick, our microspheres appear as massive opaque objects in the bright mode TEM images, as they block the transmission of the electron beam across the sample. Interestingly, we noticed that silica microspheres collapsed after few seconds of observation under the effect of the incident 200 kV electron beam, as shown in Fig. 5(B). Such a collapse shows indirectly that the microspheres should exhibit a hollow soft-like structure. The observed collapse mechanism may be explained according to the following scheme. Under the effect of the incident electron beam, the outer surface of the microcapsules becomes negatively charged, which in turn should induce, by influence, a net positive charge on the inner surface of the microcapsule, creating hence a net electric polarization across the shell. Assuming for instance an asymmetric distribution of charges between the upper region of the microcapsule and its lower region may explain the appearance of a net electrostatic force between the lower and the upper regions of the microcapsule, which may be responsible for the collapse of such microcapsules. More interestingly, Fig. 6 shows that the induced electrostatic forces may cause also the merging of neighboring microcapsules.

**Figure 7:**
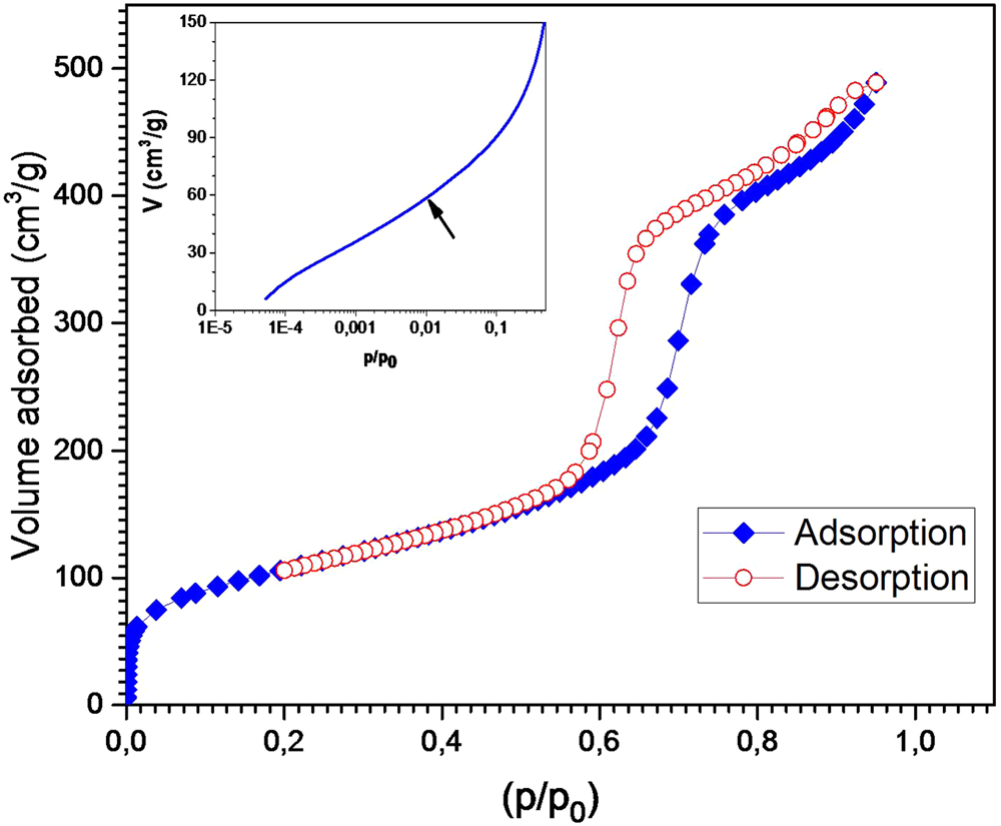
BET adsorption/desorption isotherms of mesoporous silica microspheres.

In order to determine the porosity and mass density of our microcapsules, we performed BET (Brunauer, Emmett, and Teller)^18^ measurements, with nitrogen N_2_ as adsorbate. This technique is routinely used for the characterization of porous materials with mesopores (2–50 nm diameter range) as well as with micropores (diameter <2 nm). A typical Nitrogen adsorption-desorption isotherm diagram recorded from our samples is shown in Fig. 7. According to IUPAC^19^, the observed isotherm diagram is of type IV. This indicates the presence of cylindrical mesopores with bimodal pore openings, i. e., pores are open at both ends^20^.

BET adsorption isotherm shows a steep rise in the low-pressure region, $\frac{p}{{p}_{0}} < 0.5$, and the presence of an inflexion point at $\frac{p}{{p}_{0}}\simeq 0.01$, as emphasized in inset of Fig. 7. These two features could be attributed to the presence of micropores (size <2 nm) in the sample^21^. The pore-size distribution calculated from the adsorption isotherm (not shown) is comparatively narrow and has a distribution maximum at a pore diameter of 5.9 nm. The specific surface area of our MSMs averaged over several production runs was found about *S*_*sp*_ = 514 *m* ^2^/*g* with a cumulative pore volume of *V*_*pores*_ = 0.76 *cm* ^3^/*g*, (see Table 1). One may note that the calculated surface area of the microspheres (diameter ≃10 *μm*), which is about 1.1  *m* ^2^/*g*, can be neglected by regards to the internal surface area (514  *m* ^2^/*g*) of the microspheres.

**Table 1:** Textural parameters of glasses, Specific surface area (S), volume occupied by pores (V_*pores*_) and pore size (D_*pore*_), synthesized by microfluidic-assisted sol gel method.

Specific surface	Specific pores volume	Pores diameter
S = 514 (m^2^/g)	V_*pores*_ = 0.76 *cm* ^3^/*g*	D_*pore*_ = 5.9 (nm)

**Figure 8:**
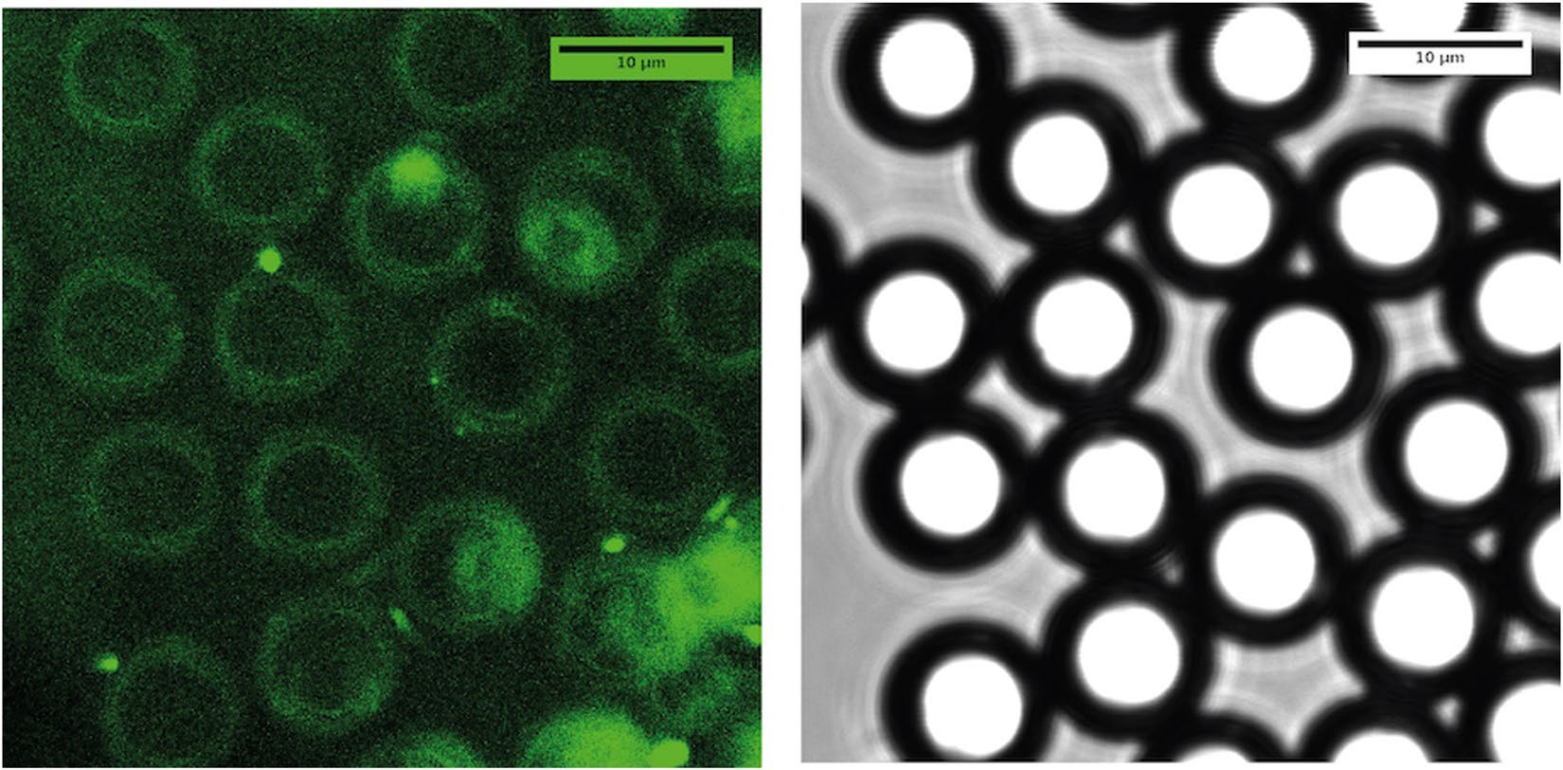
Fluorescence (left) and white field (right) confocal microscopy images of the same ROI of silica microspheres sample doped with fluorescein. These images show that the 30 *μm* silica precursor microdroplets lead to 10 *μm* silica microspheres whose shell thickness can be roughly estimated from fluorescence images (*h* ~ 1 *μm*).

Also, in order to confirm the hollow feature of our microcapsules, we doped sol droplets with fluorescein and analyzed the synthesized silica microspheres using fluorescence confocal microscopy, as shown in Fig. 8. Images shown on Fig. 8 demonstrate clearly that silica forms a thin shell around the microspheres. However, though fluorescence image does not allow to measure accurately the thickness *h* of the silica shell, one may get a rough estimation of its value, *h* ~ 1 *μm*.

**Figure 9:**
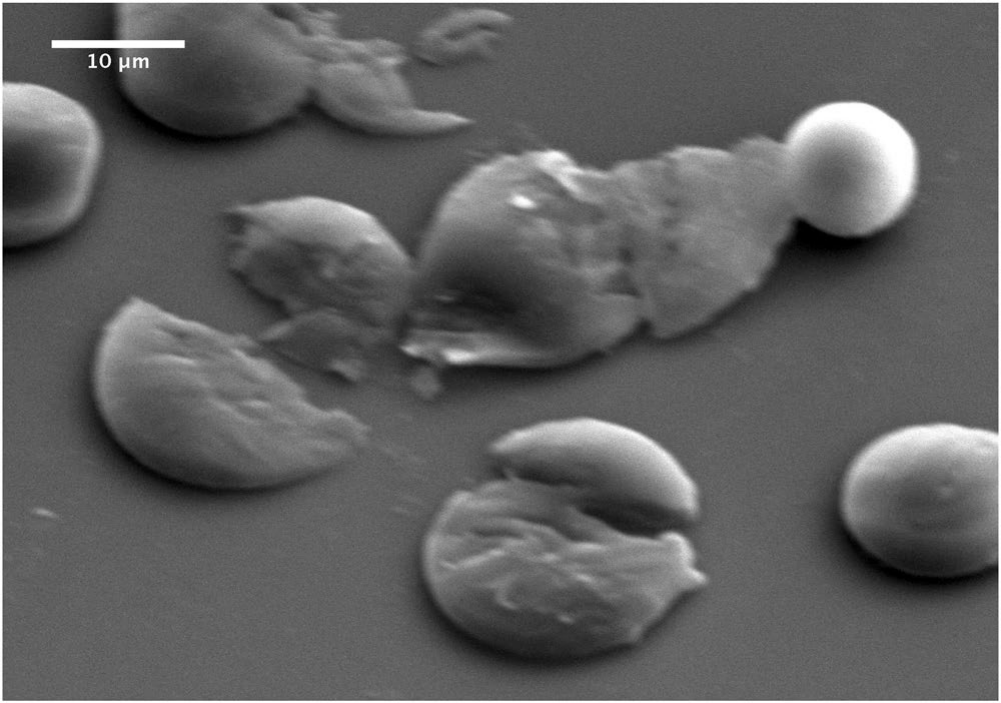
SEM micrograph of partially desiccated silica microspheres after their collapse following an abrupt depressurisation in the vacuum chamber of the scanning electron microscope. This image confirms the hollow feature of the silica microspheres and give a shell thickness value *h* ~ 0.5 *μm*.

A more accurate method for the measurement of the shell thickness *h* would have consisted on breaking the silica microspheres and measuring the thickness of the resulting fragments, using SEM or TEM image analysis. Despite the use of standard fragmentation techniques which employ either mechanical grinding or cryo-fracture, the shell rapture could not be achieved because of the elastic nature of microparticles. we succeeded to explode the microspheres by submitting the samples to an abrupt depressurization in the vacuum chamber of the surface scanning microscope (after a mild drying at room temperature during 24 hours). Figure 9 shows that this action leads indeed to the explosion of some silica microspheres, whose image analysis allows to give a more accurate shell thickness, *h* ≃ 0.5 *μm*.

Basically, one may calculate also directly the shell thickness value by taking into account BET data and the silica mass conservation inside microdroplets, as we detail in the following.

Since the massless pores occupy a specific volume V_*pores*_ = 0.76 *cm* ^3^/*g* (per mass unit), 1 g of silica should occupy a volume ${V}_{Si{O}_{2}}=\frac{1}{\rho (Si{O}_{2})}=0.45c{m}^{3}/g$, where $\rho (Si{O}_{2})=2.2$ *g*/*cm* ^3^ stands for the standard mass density of non mesoporous silica. Thus, the overall volume occupied by both silica and pores per mass unit should be equal to ${V}_{si{o}_{2}}$ + V_*pores*_ = 1.2 *cm* ^3^/*g*.

Consequently, the mass density of the silica shell, ${\rho }_{shell}$, may be deduced:

3 $${\rho }_{shell}=\frac{1\,g}{1.2\,c{m}^{3}}=0.83\,g/c{m}^{3}$$

One may deduce also the thickness of the silica shell, *h*, according to:

4 $$h=\frac{{m}_{Si{O}_{2}}}{{\rho }_{shell}4\pi {R}^{2}}=1.1\,\mu m$$

where ${m}_{Si{O}_{2}}=2.9\times {10}^{-8}$ g represents the mass of silica contained in a single microsphere; ${m}_{Si{O}_{2}}$ value is calculated by considering the used TEOS concentration (0.34 M) and the microdroplet radius (R = 15 *μm*). It’s interesting to note that the calculated value of the shell thickness is in a good agreement with the estimated value from SEM and fluorescence microscopy images. Worth noting, the measured value of thickness from SEM measurements was found almost half the calculated value. This is may be explained by the fact that the shell has been probably extended and thinned under the effect of the depressurization in the vacuum chamber before the microcapsules collapse.

**Figure 10:**
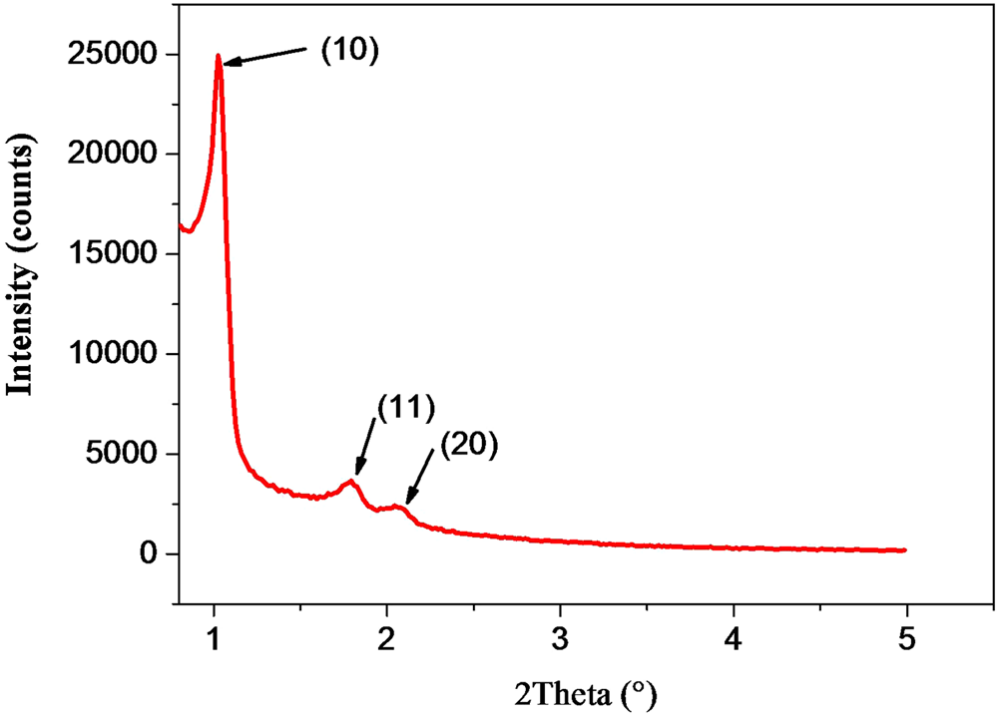
Small angle x-ray diffraction pattern showing the well-ordered hexagonal array of nanopores in silica microspheres.

**Figure 11:**
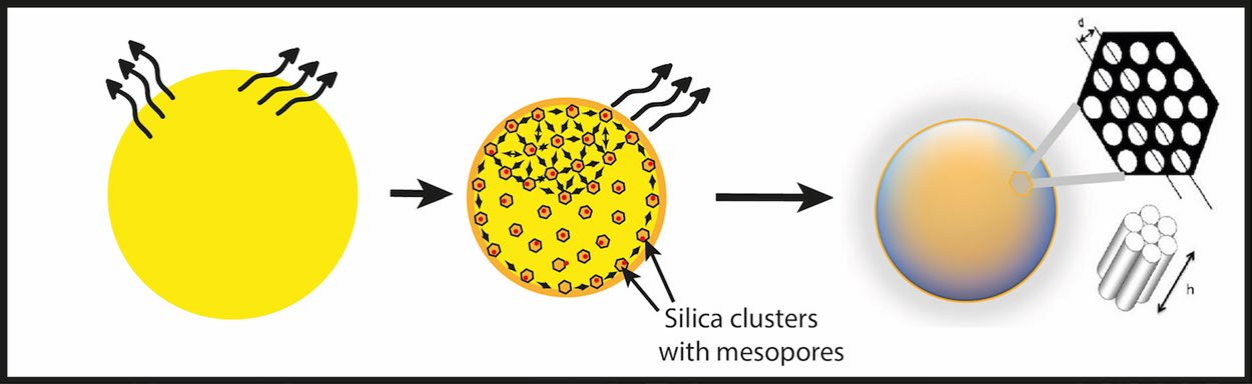
The condensation of silica precursor microdroplets leads to the formation of highly monodisperse mesoporous silica microcapsules where mesopores form a well-ordered hexagonal lattice.

Finally, in order to characterize the mesopores organization within the silica microspheres shell, we performed small angle x-ray diffraction (SAX) measurements, as shown on Fig. 10. The appearance of three significant peaks at $2\theta ={1.2}^{\circ }$, 1.8° and 2.1°, which correspond to standard hexagonal lattice reticular distances: d[10] = 8.5 nm, d[10] = 4.9 nm and d[20] = 4.25 nm, show clearly that mesopores form a well-ordered hexagonal structure, as sketched in Fig. 11. The formation of well-ordered mesopores, open at both ends across the microcapsules shell, should allow for the fabrication of microcapsules with well-controlled mass transport properties and finely tunable optical properties as well. Such features may be useful for instance for the study of whispering gallery mode resonances (WGMs) of well-defined doped microspheres and the development of highly sensitive label-free sensors and microlasers^22–24^.

**Figure 12:**
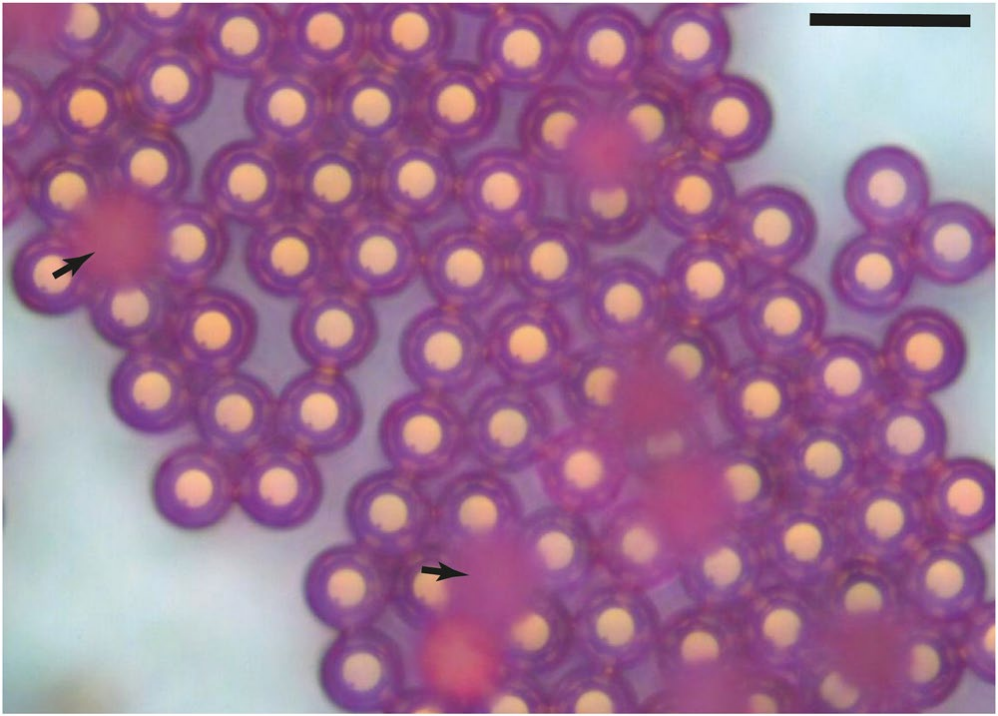
Optical microscopy image showing the swelling of silica microcapsules after they have been loaded with a rhodamine dye solution: microcapsules size increases from 10 *μm* to 20 *μm* during ~24 hours; arrows indicate floating microcapsules at the surface of the rhodamine solution, which are blurred because of their out of focus; scale bar = 40 *μm*.

Figure 12 shows one of our preliminary results where silica microcapsules have been loaded with a fluorescent dye (Rhodamine 640), after dispersion in a 1 mM dye solution during 24 hours. During this process, the size of microcapsules increased from 10 *μm* to 20 *μm*, approximately. This indicates a soft and elastic-like feature of the microcapsules shell, which allows for the adsorption of large quantities of different solvents and dyes in a controlled manner. To the best of our knowledge, this is the first report of the synthesis of highly monodisperse and mesoporous silica soft microcapsules, which compared to organic polymer-based microcapsules, should exhibit better mechanical and chemical stabilities. Also mesoporous silica microcapsules surfaces can be easily modified chemically to target some specific biomolecules. Moreover, silica microparticles exhibit a low cyto-toxicity which may open the door towards promising biological and medical applications^25,26^. A more detailed study concerning the mass transport and optical properties of doped silica microcapsules with different dyes and solvents is currently under investigation.

## Methods

Tetraethyl orthosilicate (TEOS, Si(O-EtOH)_4_) and Pluronic P123 amphiphilic block copolymer (PEG-PPG-PEG, Mn ~5,800 g/mol), which exhibits a hydrophilic lipophilic balance (HLB) ranging between 7 and 9, were purchased from Sigma and were used as a silica source and a meso-structuring agent, respectively. The silica precursor sol was prepared by dissolving 3.6 g of TEOS in a 50 ml of distilled water and ethanol mixture (50% vol/vol), 1 g of P123 polymer dissolved in 20 ml of 2 M HNO_3_ solution and 5 ml of distilled water. TEOS concentration was about 7.2% (w/w). The solution was stirred vigorously during 3 hours at 60 °C and *pH* = 2. TEOS molecules are fully hydrolyzed during this step to silanol molecules, Si(OH)_4_, according to the well-known acidic hydrolysis reaction scheme:

$$\begin{array}{l}{\rm{Si}}{({\rm{O}}-{\rm{Et}})}_{4}+{{\rm{H}}}_{2}{\rm{O}}+{\rm{EtOH}}\to {\rm{HO}}-{\rm{Si}}{({\rm{O}}-{\rm{Et}})}_{3}+{\rm{EtOH}}\\ {\rm{HO}}-{\rm{Si}}{({\rm{O}}-{\rm{Et}})}_{3}+{{\rm{3H}}}_{2}{\rm{O}}\to {\rm{Si}}{({\rm{OH}})}_{4}+{\rm{4EtOH}}\end{array}$$

The used microfluidic device for the production of sol microdroplets was prepared by standard PDMS-based soft lithography technique^27^. A flow-focusing geometry was designed with a rectangular main channel (width ≃30 *μ*m and height ≃30 *μ*m). A commercially available HFE 7500 fluorinated oil (3-ethoxy-dodecafluoro-2-trifluoromethyl-hexane, Inventec), having a density of 1.62 g/cm^3^, was used as the carrier oil. This oil does not cause PDMS swelling and does not solubilize most of non-fluorinated organic molecules, including droplets contents. Droplets were stabilized using a home-prepared copolymer surfactant, derived from a commercially available carboxy-terminated fluorinated polymer, namely Krytox 157-FSH (Dupont) and a solution of benzyl-trimethylammonium hydroxide (BTA, Sigma-Aldrich). A commercial surface coating agent (fluorosilane) dried with *N*_2_ was used in order to increase the wettability of oil phase on the channel walls. Volumetric flow rates were set to *Q*_*oil*_ = 300 *μ*L/h and *Q*_*aq*_ = 50 *μ*L/h for the oil phase and the aqueous phase, respectively, fr all experiments (Nemesys, Cetoni GmbH). In these conditions, highly monodisperse microdroplets with a diameter of about 30 *μ*m (±1 *μm*) were produced at a rate ~1 kHz.

After their production in the microfluidic device, sol microdroplets were collected in a Petri dish as shown in Fig. 2, then left at rest during 24 hours, at room temperature to fully achieve their gelification and finally were desiccated at 150 °C during 24 hours. Droplets image analysis was realized using an inverted X81 Olympus optical microscope and ImageJ. Dessicated silica microparticles were characterized using a HITACHI S-3400N scanning electronic microscope (SEM), equipped with an energy dispersive spectroscopy (EDS) chemical analysis additional device. Transmission electron microscopy (TEM) were performed at 200 kV an d room temperature using a TITAN^3^ G2 microscope.

Fourier Transform Infrared (FTIR) absorption spectra were performed using a Bruker Equinox 55 spectrometer, in the range of 400–4000 cm^−1^1, at room temperature, after mixing 1 mg of the silica microspheres with 200 mg of dried KBr.

Porosity of samples was determined using BET (Brunauer, Emmett, and Teller)^18^ measurements and a FlowSorb II 2300 apparatus, with Nitrogen as adsorbent and an analysis bath of 77 °K. Samples were degassed beforehand at 200 °C to remove the residue molecules of water deposited in the porous structure of the sample.

Small angle X-ray diffraction measurements were performed using a PanalyticalX’Pert Pro diffractometer equipped with a Cu tube, a Ge (111) incident-beam monochromator (*λ* = 1.5406 Å) and an X’Celerator detector. Small-angle X-ray scattering (SAXS) measurements were collected using 0.02 rad Soller slits, 1/16° fixed divergence and anti-scatter slits. The X’Celerator detector was used as “scanning line detector (1D)” with 0.518° active length. Data collection was carried out in the scattering angle range 0.5–6° with a 0.0167° step over 60 min.

## Conclusion

We have developed a new microfluidic-assisted method for the fabrication of highly mesoporous silica microcapsules. This method is based on the combination of the solvent evaporation method and the use of highly monodisperse droplets as soft-templates for the synthesis of well-defined monodisperse mesoporous silica soft-like microcapsules. Our approach is much simpler and straightforward than the standard technique which utilizes sacrificial hard templates. We show that the formation of the silica soft shell is driven only by the control of the balance between the solvent evaporation and the silica solidification rates at the surface of the microdroplets. Depending on both the physico-chemical properties of the precursor solution and the fabrication process, a silica soft-like shell with a thickness of about 1 *μm* and across which mesopores with a diameter of 5.9 nm form a well ordered hexagonal 2D network. We show also that the synthesized microcapsules can absorb a large quantity of an aqueous (stained) solution by increasing their size by a factor two, approximately. This confirms the opening of mesopores at both ends across the microcapsules shell, as first shown by BET measurements.
